# The Meaning of Collaboration, from the Perspective of Iranian Nurses: A Qualitative Study

**DOI:** 10.1155/2014/785942

**Published:** 2014-12-17

**Authors:** V. Zamanzadeh, A. Irajpour, L. Valizadeh, M. Shohani

**Affiliations:** ^1^Department of Medical and Surgical Nursing, Nursing and Midwifery School, Tabriz University of Medical Sciences, Tabriz 5138947977, Iran; ^2^Social Determinants of Health Research Centre, Isfahan University of Medical Sciences, Isfahan 8174673461, Iran; ^3^Department of Pediatrics, Nursing and Midwifery School, Tabriz University of Medical Sciences, Tabriz 5138947977, Iran; ^4^Department of Medical and Surgical Nursing, Nursing and Midwifery School, Student Research Committee, Tabriz University of Medical Sciences, Tabriz 5138947977, Iran

## Abstract

*Background*. Interdisciplinary collaboration among nurses is a complex and multifaceted process, an essential element in nursing, which is crucial to maintain an efficient, safe, and viable medical setting. The aim of this study was to explore the meaning of concept of collaboration through conducting a qualitative research approach. *Method*. The present study is qualitatively conducted in a content analysis approach. The data collection process included 18 unstructured and in-depth interviews with nurses during 2012-2013 in educational medical centers of west and northwest of Iran. A purposive sampling method was used. All the interviews were recorded, transcribed, and finally analyzed using a qualitative content analysis with a conventional method. *Result*. Categories obtained from analysis of the data to explain the meaning of collaboration consist of (i) prerequisites of collaboration, (ii) actualization of collaboration, and (iii) achievement of a common goal. *Conclusion*. The results of the present study ended in the discovery of meaning of collaboration that confirm results of other related studies, hence clarifying and disambiguating the concept under study. These results also contribute to the development of collaboration theories and the relevant measurement tools.

## 1. Introduction

For many people, collaboration is an essential element of work relationships in any profession [1]. Collaboration has been discussed in various disciplines including organizational behavior, management, environmental science, communications, education, sociology, anthropology, history, and medicine. Every discipline has its own particular perspective on collaboration as a form of interaction [2]. In the recent years, the concept of interprofessional collaboration in the health system has attracted a huge interest due to the impact it has on quality of care and its outcomes [3]: increased quality of care and effectiveness of treatment, satisfaction among professions in terms of professional wellbeing, reduced staff turnover, and problems associated with professional performance. These problems include increased coordination and joint accountability, creativity and patient satisfaction, reduced hospital stay and unnecessary administrative monitoring, improved medical management outcomes, more holistic patient education, and prevention of medical errors [4, 5]. Many studies have cited poor collaboration among care providers as one of the most common and important causes of medical errors [6–8]. Poor collaboration and communication was also reported as the origin of all inadvertent accidents [9]. Accordingly, providing health care services requires collaboration of health care workers among various medical and paramedical disciplines and departments [10].

There is an extensive literature on health service management and nursing and to a lesser degree on collaboration within medical practitioners. Reviews show that studies carried out on this subject are related to interactions between physicians and nurses [11], limited to specific departments like special care units, emergency, and oncology [12, 13]. Collaboration and teamwork are considered as two essential elements in nursing [14]. Therefore, increasing collaboration among nurses to maintain an efficient, safe, and viable medical setting is crucial [15]. Prior to the past decade, a need for new models of collaboration has been felt, to provide services by nursing teams [16].

Various nursing associations have proposed standards for nursing practices, where the need for collaboration among nurses has been cited as an essential element. Collaboration is a quality that all nurses are expected to possess; however, interdisciplinary collaboration among nurses is a complex and multifaceted process that does not occur spontaneously, and several factors contribute positively and/or negatively [12].

Collaboration is a complex and multidimensional concept that is defined in different methods [17]. The term “collaboration” is originally a combination of the Latin prefix “co” meaning togetherness and the morpheme “laborate” meaning to work [18]. Oxford Advanced Dictionary defines the term as “working with another person to produce something” [19]. It has been defined in Merriam-Webster Dictionary as “working jointly with others, or together, especially in an intellectual effort” [20]. Conceptually, the term collaboration has been defined differently by various researchers. Some believe that collaboration should inherently be understood and not defined [21]. Some regard it as an outcome [22] that results from synthesis of different views [23]. In many studies, it has been defined as a process [23–25], special process or processes that occur during interactions. Some have focused more on collaboration as a structure, but some have failed to clarify whether it is a process or a structure at all [2].

There are numerous definitions derived from the theories and frameworks prevailing among professions [26]. This has marred a clear understanding of the meaning and an accepted general definition of the concept [27]. The unanimous disagreement on one clear-cut definition of the concept has led to emergence of different approaches both in research and in practice [28]. Therefore, the concept and its variant meanings in the literature have brought about misunderstanding among the practitioners in the field, the authorities and healthcare workers, and hence no common shared understanding [29]. In many cases, the concept of collaboration replaces other concepts such as teamwork, partnership, coordination, communication, and cooperation, while the meaning and intentions in any case are conceptually different [2, 4].

Given that healthcare system is different from other professions and effective collaboration is context dependent [30], the meaning ought to be empirically derived from understanding of those who participate in the study [4]. Investigating this concept through a qualitative research, which includes multiplicity of data collection procedure and an event examination method, norms and values from the participants' viewpoint, provides the possibility of an in-depth examination, clear and comprehensive understanding, and recognition of the phenomenon [31]. The result of the study is essentially fruitful in the context of the Iranian healthcare system. The importance of a common definition shared among the professionals and the lack of qualitative research on the concept of collaboration within the Iranian cultural and linguistic constraints motivated this research study to be carried out.

## 2. Methods

### 2.1. Design and Participants

A qualitative research design was implemented to explore the meaning of collaboration and its inner dimensions as understood by the nurses. Considering the nature of collaboration and in order to reach a comprehensive perception of the concept in question, participants were selected from among nurses currently employed at teaching hospitals. Eighteen participants who participated in the study included one nursing service manager, one triage supervisor, two head nurses, three PCTs, eleven nurses (some of them employed under temporary contract and others were employed permanently) working in emergency, internal, surgery, hemodialysis, post-CCU, and ICU (lung, general medicine, and surgery) wards with a minimum one-year work experience.

### 2.2. Data Collection

In this study, a purposive and a theoretical sampling method was used. After obtaining the informed consents, data were collected through some in-depth, unstructured interviews with the participants. The interviews were digitally recorded, typed verbatim on the same day, and used as the main source of the study data. These interviews were conducted in one or two consecutive sessions in a private room, in the participants' workplace at teaching hospitals of Tabriz, Tehran, and Ilam Universities of Medical Sciences. Each interview lasted between 30 and 90 minutes. The entire data collection phase began in April 2012 and ended in February 2013.

### 2.3. Data Analysis

A conventional content analysis was used to analyze the collected data. To this end, each interview was typed immediately after interview session. They were then transcribed, read, and reviewed several times. Next, the transcripts were divided into meaning units in sentence and paragraph frame within which the related main meaning occurred. The meaning unit identification was repeated several times and the appropriate codes were then written for each of them. The codes were then categorized according to conceptual and meaning similarity criteria. The declining trend in data reduction was present in all analysis units, main categories, and subcategories. Finally, the data were placed in the main categories which were more general and the main theme was abstracted at the end. With the addition of each interview, the analysis process was repeated in the same way and the relationship between them was found and sampling continued until saturation of data (repeated data and identified characteristics of the concept).

### 2.4. Trustworthiness of the Study

During the study, specific methods were used to ensure viability of the data; four criteria, according to Guba and Lincoln (quotes by Polit and Beck) [32], including credibility, verifiability, reliability, and transferability were taken as the frame of reference. Moreover, member check was used in addition to prolonged involvement of the researcher to increase the credibility of the data. Also, after encoding, the interview transcripts were returned to the participants to ensure the accuracy of the codes and the relevant interpretations. Peer check approach was used to control for data confirmability; for this purpose, the data were coded and categorized which were later evaluated by the research team. When between codes and categories there was no consensus, discussion was continued to clarify the issue and to reach a consensus. To control for the dependability of the data, an audit trail was used. In implementation of this method, the researcher maintains the preliminary data, categories, and themes until the end of the research process. Moreover, the review and analysis of the data of the experienced individuals in the research team increased the reliability of the study. The transferability of the study also depended on the evaluation and approval of findings of the individuals in the same environment. Sampling with a maximum variance also helped in the transferability and stability of the results as well as credibility of the data [33]. Allocation of sufficient time to the study and face-to-face communication with the participants was another factor that increased the data credibility. Results were also confirmed by some nonparticipant nurses.

## 3. Results

Eighteen Iranian nurses, four male and fourteen female, thirteen of whom were married and aged between 22 and 44 years (mean: 32.4 ± 7.3), were interviewed. Among them four people hold a high school diploma, while fourteen hold a bachelor's degree whom had been graduated from a four-year baccalaureate program. Their clinical experiences ranged from two to 25 years (mean: 10.4 ± 6.9) in specialized areas of emergency, internal, surgery, hemodialysis, post-CCU and ICU care. Data analysis revealed three main categories: (i) prerequisites of collaboration; (ii) actualization of collaboration; and (iii) achievement of a common goal. These categories and their subcategories are presented in Table 1.

## 4. Prerequisites of Collaboration

From this category, eight subcategories emerged: (i) understanding, (ii) friendship and sincerity, (iii) commitment and accountability, (iv) satisfaction, (v) team making, (vi) communication, (vii) trust, and (viii) respect.

### 4.1. Understanding

An issue cited by the participants was awareness of and knowledge about each other and activities of the colleagues in other wards in order to help them. One of the participants in this regard said: “When I help a colleague, I have understood her situation, the ward, the patient, and the patient family” (P.1).

Based on the findings, “understanding” will not be limited to a colleague, but the patient condition is also a priority. Another participant said: “Understand the situation, understand the colleagues, and the most important understanding is the patient and their family. We must understand the patients and their families” (P.2).

### 4.2. Friendship and Sincerity

This subcategory plays such an important role in how nurses work together which it is one of the essential prerequisites for helping others without their demand. Participants have frequently mentioned friendship among team members. One of them (P.1) said: “When my friend is sick or has a problem, or has a critically ill patient, I see him not being able to do his other duties; I do them without telling him. We are somehow friends with each other.”

Another participant said: “… my head nurse tells us try to be friends with each other. Trying to keep together” (P.3).

In fact, friendship and intimacy between participants led to a problem solving process and job performance accelerated as a result. However, they mention this issue not only at the workplace environment but also their friendship continue even outside of workplace.

### 4.3. Commitment and Accountability

Commitment and accountability to a group and to work deposited in a person is an important feature that can be inferred from the participant's statements.

A participant argued: “When I help my colleague, and someone else doesn't, it means she isn't accountable, and isn't committed. I expect her to show greater participation in the work of the ward” (P.9).

A participant (P.18) referred to mutual responsibility as strong collaboration and said: “Strong collaboration is that other colleagues in other units feel as much responsible as I feel toward my work and patients.”

### 4.4. Satisfaction

Satisfaction is another essential prerequisite in collaboration with others and demands participation of every team member. A participant stated: “To help each other, both sides should be content to do so” (P.16).

A participant (P.17) said: “Being satisfied with each other is a reason for working with each other” and another one (P.15) said: “It may happen that someone will not be satisfied when you help them.”

The entire quotes mentioned above imply that collaboration required mutual satisfaction of the participants to recognize and verify their collaboration and sharing of scientific and practical experience.

### 4.5. Team Making

Participants considered themselves as team players and clearly stated: “In our profession, working alone is impossible, and it must be done in teams” (P.2).

To enter the team and help it succeed, participants obligate themselves to possess qualities like knowledge, mutual understanding, sacrifice, and self-devotion for preserving the group's solidarity. One of them (P.18) in this case said: “Having the knowledge and the ability to cope with the hardships were some of the factors for entering into the team. In this team, sometimes we had to put aside what we had. For example, I came at a fixed time in the morning and worked in the clinic in the afternoon. Then a colleague had a problem and the head-nurse asked me to come at a fixed time in the afternoon. Since I wanted to preserve the team, I left the private work in the clinic and came to the work place as I was asked to.”

These factors lead to the maintenance of a team and its success.

### 4.6. Communication

Communication between health care providers to provide safe and effective care is important.

Proper communication leads to dialogue and information exchange among team members would be as a result an advantage to problem solving.

“I think the first moment is important, when a new person joins the group, and we should establish a membership contact with her” (P.13) said a participant.

Despite the importance of communication resulted from the participants experiences in this study, there were challenges in communication among them. These challenges not only created problems in providing the patient with care but also created tension for the nurses as well.

### 4.7. Trust

This feature is frequently cited in the field which signifies its importance. It is considered as a quality among colleagues that their scientific and practical competence to perform the tasks would be enhanced if they show any degree of this quality. In their statements, participants cited such cases as communication, awareness and knowing each other, friendship and togetherness, and time taken to develop trust between them. According to one participant, “We had assessed and tested each other, and trusted each other, and knew how competent the other one is” (P.15).

Another participant stated: “As time goes by, people trust each other, because they get to know each other and know their personality more” (P.14).

### 4.8. Respect

Mutual respect and value are essential; according to a participant, “Interpersonal respect is created by oneself. When I meet expectations of the person in charge of the ward, I only expect respect in return, not giving caution to me for the smallest things in the presence of others” (P.11).

Mutual respect is essential to work in teams and in collaborating with others. This includes respect for everything from professional character to the abilities of all team members.

## 5. The Implementation of Collaboration

The participating nurses' perception of implementation of collaboration encompasses two subcategories: (i) pragmatism and (ii) joint decision-making.

### 5.1. Pragmatism

Based on the requirements identified for collaboration from the participants' statements and based on the subcategories of pragmatism, they have pointed out some issues that imply the way they collaborate with each other. They have mentioned supporting each other according to an understanding of the priorities of tasks in their statement. One of the participants in this regard said: “When my colleague's patient is sick, and I can postpone my own work, I do my colleague's work first, and support the colleague that really needs urgent help” (P.8).

In the participants' statements, work prioritization is observed in two forms: Some people give priority to the work they have been assigned to, and they help others according to their needs afterwards. And others give priority to their colleague's work, while in crisis, for example, when a patient is being resuscitated and when a colleague is unable to do their work because of a heavy workload, fatigue, or illness. In this respect, participants cited cases of covering and supporting each other based on an understanding among them. Voluntary help was among important characteristics mentioned by the participants in the present study. In some cases, a person may ask for help and get positive response, while in others people are helped without asking for it. A participant as a piece of evidence to this statement (p.18) said: “If it is about my crony, I will go to help without asking them and if they do not want to, I will go voluntarily.”

In relation to “give-and-take” characteristic at work, which means expecting compensation for the help you have done, some present study participants considered it a “rule at work,” and some considered helping others a duty and did not expect anything in return. A participant said: “In working together, there is a trade-off between us” (P.7).

### 5.2. Joint Decision-Making

In our participants' statements, joint decision-making among nurses of a ward was considered important for providing better patient care, which in turn depends on the level of nurses' expertise and the character of the patient. A nurse (P.17) in this case said: “Most of the nurses here are expert and do their job, but in certain situations, such as when the patient is critically ill, joint decision-making is put into practice.”

However, as far as doctors are concerned, as a group having maximum contact with clinical nurses, no joint decision-making is seen, and physicians make decisions alone and issue orders, and nurses should obey them and provide information only. For instance, a participant argued: “A doctor has ordered an ECG; “is it necessary,” I asked? “Just do it” said the doctor. The participant continued: “afterwards, he doesn't even look at it” (P.18).

## 6. Achieving a Common Goal

### 6.1. Client Centered

During the research period, it was also found that participants' efforts were all directed toward a common goal, efficient patient care, an issue cited by participants, as giving priority to patient in working together; a participant (p.6) said: “collaboration means efficient functioning for patient health…” and another one (p.16) declared: “our goal is to strive for better and perfect service for the patients.” In another statement, a participant remarked: “lack of collaboration hurts the patients” (p.6).

Since the satisfaction and wellbeing of the patient was considered as a common goal, all these quotes represent attempts to maintain patient safety.

## 7. Discussion

This study aimed to explore the meaning of collaboration from the perspective of Iranian nurses; the results of their experiences indicated the actualization of collaboration and its properties that are observed in real life situations. In the field study, it was found that the participants considered collaboration as an important and influential potential in their work, with subsequent positive outcomes such as providing a safe care, with reduced error and mutual satisfaction of the personnel and patients. According to their definition of the concept and benefits of working together, they reported that they back up each other in the work place, helping each other to work efficiently. Intellectual and practical partnership, voluntary help, give and take at work, and satisfaction obtained from reaching a common goal were among the stated merits of collaboration. In studies like the present one, similar cases have been cited in defining collaboration; for instance, in a qualitative study by Moore and Prentice on collaboration between NP and RN nurses, “togetherness” in the form of “spending time together inside and outside clinic, and face-to-face interaction” was one of the themes that accelerated collaboration [12], a trait also reported by the participants in the present study. In a wide range of specialties, togetherness creates opportunities for providing better services, which complements benefits to patients [14].

Participants in this study contend that understanding among nurses themselves besides an understanding between the nurse and the patients was very important. Furthermore, understanding colleagues' roles was a vital step toward collaborative participation. This last quality requires sufficient time for team members to get to know each other's skills, needs, and unique responsibilities [34]. Healthcare professionals that have understood mutual roles are able to work effectively together, providing higher quality of care [35].

Teams need to have commitment. In this study, participants were aware of ethical and work commitments required for their job that indicated some kind of personal accountability. They considered it a value, while lack of commitment prevents the development of collaboration among the members. In a study conducted by Daniels and Khanyile, a theme obtained was lack of commitment to begin collaboration [24].

To provide safe team care, satisfaction is a principle. In this regard, Hodges et al. state that collaboration requires participants' consent to identify and confirm their relationships and to share risks, resources, accountability, and rewards. In the literature, intellectual and practical actions [11] are referred to as an attribute of collaboration.

As mentioned by the participants, teamwork is recognized as a factor for maximizing clinical efficacy and depends on interdisciplinary collaboration of relevant professions [36]. Deming believes that teamwork is specific to a system, with all its staff working well toward achieving a common goal [37]. Despite the fact that interdisciplinary collaboration requires teamwork, Jansen points out that collaboration is often lost in the context of team [38].

In many studies, communication is considered an attribute of collaboration [39], and collaboration requires extended communication in order to achieve goals that cannot be achieved alone [40]. Healthcare system also requires nurses that are able to establish successful communication with multidisciplinary team members, patients, and their families [41]. The present study participants stated that there is a good communication among team members including nurses with each other and with patients. They also believed that it begins as soon as one enters the ward. Despite an awareness of the importance of communication, errors that occur or missed care is caused by poor communication [35], which also existed between nurses and physicians in the context of the present study. A study by Sasahara et al. on “collaboration among nurses” showed that in 16% of nurses' statements that there was little opportunity for discussion between nurses, 10% of nurses were unable to share their feelings with other nurses, 7% of nurses were unable to provide regular and coherent services due to different attitudes in nursing personnel, and 5% of nurses had revealed poor communication among nurses [42].

Trust in each other was one of the important results drawn from the data analysis of this study. As a belief, members expect each other to perform well [43], and trust is the basis of cooperation among humans and a foundation for creating social organizations [44]. Trust must be created [45]. These results indicated earlier in the present paper are similar to those of a study by Moore and Prentice, in which one of the themes was length of time of being together and subsequent trust and respect [12].

In addition, another important feature that was mentioned by the participants is respect. Respect for and trust in others' decisions and skills in a team require full understanding of different professional perspectives involved in that team. Our professional behavior and life should contain observable indications of respect but information obtained from historical, gender, and funding impacts showed that professional hierarchy in healthcare system has caused “turf wars,” which has led to mutual disrespect and distrust [46].

In addition to the cases discussed so far, prioritizing work based on the patient condition was one of the main issues as mentioned by the participants. Sicotte et al. study showed that degree of interdisciplinary collaboration is closely related to type of patient receiving services, so that there was greater collaboration among colleagues with patients requiring complex care [47]. In the literature, prioritizing work is discussed as an assertiveness and cooperation. Heatley and Kruske argued that collaboration involves a combined assertiveness and cooperation effort. Assertiveness means meeting one's own needs in a group, without giving priority to needs of others, while cooperation means meeting other people's needs [39].

Although collaboration is considered a kind of integration that is achieved through mutual and voluntary arrangement [48] and there is optional collaboration in organizations that enjoy high level of motivation [49], in a literature review, similar item could not be found. Systematic compensation of collaborative actions is a requirement for effective implementation of rules in collaborative environments [45]. Boone et al. also argued that collaboration involves bilateral trade-off [23].

Effective decision-making in a healthy clinical setting depends on communicational skills. Nurses should discuss the nature of relationships with patients, physicians, other care providers, and nurses themselves [22]. There is an emphasis on joint decisions and intergroup processes in interprofessional teams [50]. This is the best view toward care, which enables professions to openly discuss issues [51]. Research results have shown that nurses and residents in patient care units cannot communicate with each other without arguing and frequently disagree on patient care programs [52, 53]. Patient care priorities may be regarded differently from the health system perspective and that of the team members' [54]. Perhaps the biggest conflict between physicians and nurses is about roles to play and degree of accountability of the personnel and possible effect on patients' condition, resulting in conflict in the care objectives [55]; nevertheless, hierarchy is a common barrier to effective communication. In health care settings that are recognized by their culture of hierarchy, physicians are at the zenith [54], while the actual objective of a collaborative action is to provide a comprehensive care in any environment that meets care needs of a particular population, which is achieved through effective and full use of knowledge and skills of care providers involved in teamwork [56]. Weakness in this area is much highlighted in the context of the present study.

In literature review, collaboration is broadly identified as working with others toward achieving a common goal [22, 39, 57]. Partners should agree upon common goals for working together and partnership. Heatley and Kruske study on maternal care professions, where achieving healthy outcome for mothers and infants was considered their common goal [39]. Although focusing on a common goal is part of a collaborative approach [58] that enhances use of different pretexts [12, 59], what was observed in the present field study was a common goal among nurses in a ward and not among different disciplines, like physicians. Moreover, participants in this study considered patients as a team member and they assume respecting their rights as their duty. However, in the literature, interestingly, less attention has been paid to recipients or those who benefit from the results of collaboration [20], and no one has made a serious effort to explain how patients are supposed to integrate into the care team. Despite the fact that patients are the pinnacle of collaborative care [12], maximum involvement of patients and their families is a key part of an interdisciplinary collaboration and patient-oriented care is the basis of the interdisciplinary care, which the Canadian Ministry of Health describes as collaborative action in patient-oriented form [29].

## 8. Conclusion

It is clear that collaboration is a key strategy for improvement, problem solving, and innovation in the health system; therefore, a critical first step is developing a clear understanding of it as a concept and understanding its characteristics, which is what the participants in this study mentioned based on their experiences in a real context. The results of this study confirm the results of other studies which can help make the concept clear and disambiguate it. Themes and subthemes obtained can provide health workers, service providers, policy makers, and teachers with a broad vision to overcome obstacles of collaboration, leading to a more fruitful nursing profession and opportunity for providing better practice. Study results can be used to develop collaboration theories and measuring tools.

## 9. Limitations

As in other qualitative studies, one of the limitations of the present study is generalizability of results. Accordingly, maximum effort was made to improve rigor of data. Restricted field of study to teaching hospitals was another limitation, and it is recommended that, in future studies, experiences of people from nonteaching hospitals should be considered.

## Figures and Tables

**Table 1:** The process of obtaining theme from categories, subcategories, and codes.

Codes	Subcategories	Categories	Theme
Mutual Understanding, the necessity of colleagues understanding, put yourself in the shoes of others (colleagues, patient and their family), and understanding the difficult situation of the patient	Understanding	Prerequisites of collaboration	Collaboration
Sensible and logical friendship, the togetherness in the workplace and outside the workplace, and working together with each other	Friendship and sincerity		
Lack of commitment and not doing the patient work, commitment to the job, and responsibility and accountability for maintaining the group's ethical and legal commitment	Commitment and accountability		
Mutual satisfaction for helping each other, the necessity of satisfaction for accepting others help, and satisfaction as a reason to work together	Satisfaction		
Belief in the teamwork, togetherness, sacrifice, and devotion to keep the group	Team making		
Good communication matron, communicate with a new person, nonverbal communication, verbal communication, and correct and respectful communication with colleagues	Communication		
Trust together in helping each other, escrow things together and make sure they are done right, and trust is based on knowledge and practice	Trust		
Interpersonal respect: respect for other colleagues and respect for the patient	Respect		
Prioritize tasks and help others to help in getting things done, help each other to do extra work, job sharing, the need for mutual collaboration, and meet the mutual expectations, interchange	Pragmatism	The implementation of collaboration	
In critically ill patient, help others according to the difficult conditions of the partner, share the tasks performed by the partner, the type of care, and patient needs, share knowledge, expertise, and experience	Joint decision-making		
Emphasize the treatment of patient, improve the patient improvement, do the patient care and avoid problems and errors, providing patient safety, the patient is important for me, patient health is the first priority	Client centered	Achieving a common goal	
